# Nutritional metabolic dysregulation in T2DM as a catalyst for osteoarthritis pathogenesis

**DOI:** 10.3389/fnut.2025.1676056

**Published:** 2025-09-29

**Authors:** Chengyan Wang, Xiaodong Yu, Xianqiu Zeng, Ming Li, Wenxiao Yang, Shu Zhou

**Affiliations:** ^1^Department of Ultrasound, Jilin Cancer Hospital, Changchun, Jilin, China; ^2^Department of Anesthesiology, Jilin Cancer Hospital, Changchun, Jilin, China

**Keywords:** type 2 diabetes mellitus, nutritional metabolic dysregulation, inflammation, obesity, osteoarthritis, nutritional interventions

## 1 Introduction

Type 2 Diabetes Mellitus (T2DM) is a chronic metabolic disorder characterized by elevated blood glucose levels resulting from insulin resistance and relative insulin deficiency. It is one of the most prevalent endocrine disorders worldwide, driven by a combination of genetic, environmental, and lifestyle factors (1, 2). T2DM is associated with a range of systemic complications, including cardiovascular disease, neuropathy, nephropathy, and retinopathy, which significantly impact patient quality of life (3, 4). The pathogenesis of T2DM involves complex metabolic dysregulation, notably disturbances in glucose and lipid metabolism, often exacerbated by poor dietary habits, sedentary lifestyles, and obesity (5–7).

Osteoarthritis (OA) is a degenerative joint disease characterized by the progressive breakdown of articular cartilage, subchondral bone remodeling, and synovial inflammation (8). It is the most common form of arthritis, primarily affecting weight-bearing joints such as the knees, hips, and hands (9). OA leads to pain, stiffness, reduced mobility, and diminished quality of life, imposing a significant socioeconomic burden. Recent research recognizes the role of metabolic and inflammatory processes in its development (10, 11).

Emerging evidence indicates a significant link between diabetes and osteoarthritis (OA), suggesting that metabolic disturbances inherent to diabetes may contribute to joint degeneration (12, 13). The association is multifaceted, involving systemic inflammation, oxidative stress, and altered cellular metabolism within joint tissues (14, 15). Hyperglycemia and insulin resistance can induce inflammatory pathways that exacerbate cartilage breakdown and impair repair mechanisms (16). Additionally, diabetes-related metabolic changes, such as dyslipidemia and mitochondrial dysfunction, can directly affect chondrocyte viability and function (17). Obesity, a common comorbidity of diabetes, further amplifies joint stress and inflammatory mediators, accelerating OA progression. Recent research highlights that early-life hyperglycemic environments may epigenetically predispose individuals to increased susceptibility to OA later in life, partly through mitochondrial impairment and disrupted cellular homeostasis in cartilage (18). Understanding the intricate relationship between diabetes-induced metabolic abnormalities and joint health is essential for developing integrated therapeutic strategies aimed at preventing and managing osteoarthritis in diabetic populations.

## 2 Key factors contributing to nutritional dysregulation in T2DM

Nutritional metabolic dysregulation refers to the disruption of normal metabolic processes resulting from imbalances in nutrient intake, absorption, and utilization. It encompasses a spectrum of metabolic disturbances characterized by abnormal glucose metabolism, lipid abnormalities, and inflammatory responses. In the context of T2DM, this dysregulation manifests as impaired insulin signaling, increased hepatic glucose production, and dyslipidemia, which collectively contribute to systemic metabolic imbalance (19, 20). Such disturbances not only impair energy homeostasis but also set the stage for chronic low-grade inflammation and tissue damage, thereby influencing the development and progression of comorbid conditions such as osteoarthritis. Several interconnected factors drive nutritional metabolic dysregulation in individuals with T2DM. Central among these is insulin resistance, where cells become less responsive to insulin, leading to elevated blood glucose levels (21, 22). Excessive caloric intake, particularly diets high in refined carbohydrates and saturated fats, exacerbates this resistance and promotes adiposity (23). Obesity, especially visceral fat accumulation, acts as both a cause and consequence of metabolic disturbances, secreting pro-inflammatory cytokines that further impair insulin sensitivity (24). Additionally, altered lipid metabolism results in elevated triglycerides and low HDL cholesterol, compounding the metabolic imbalance (25).

## 3 Pathophysiological mechanisms linking T2DM and osteoarthritis

### 3.1 Inflammation and its role in OA with T2DM

**Figure 1:**
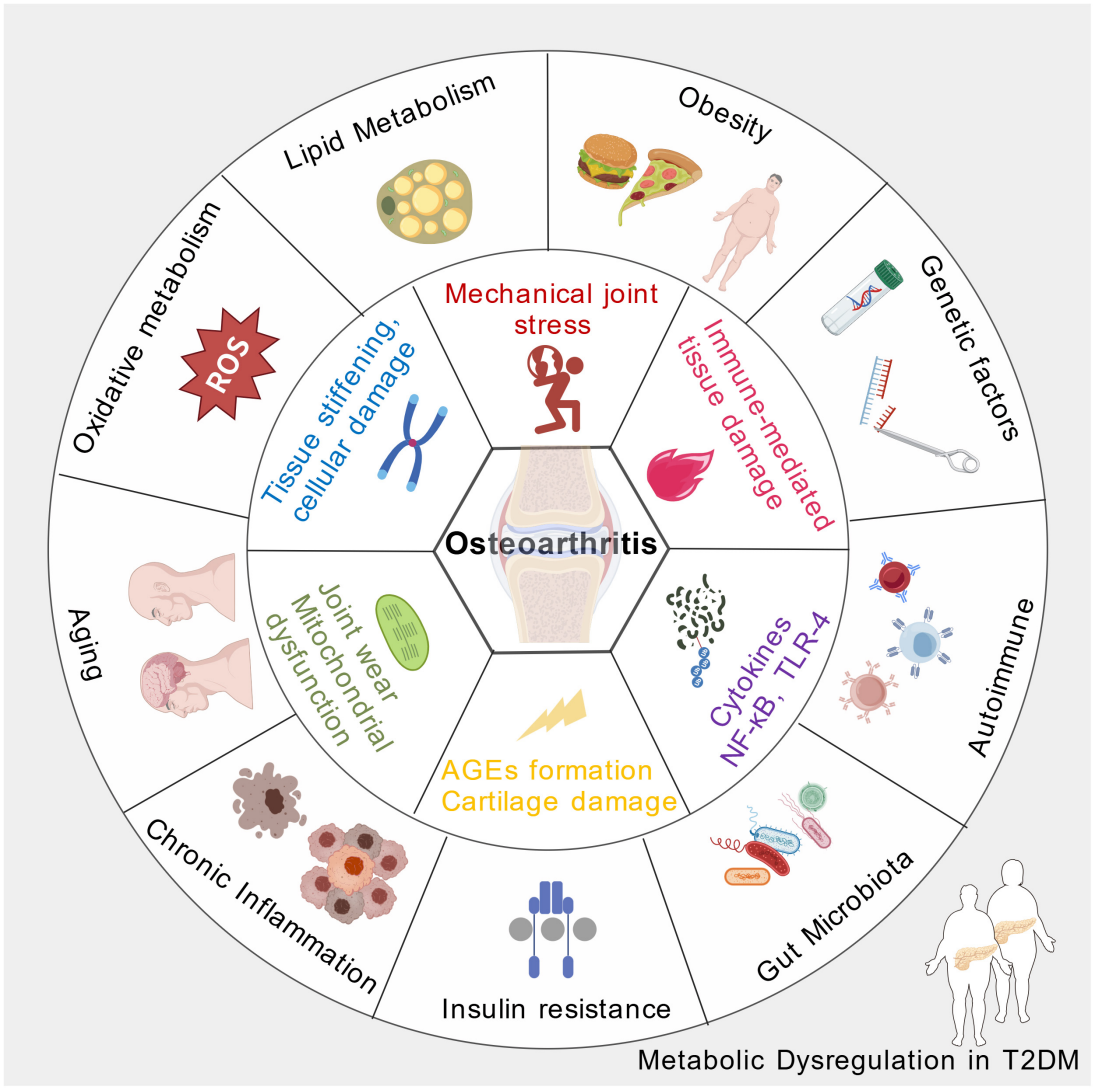
The pathophysiological mechanisms of osteoarthritis induced by nutritional disorders secondary to type 2 diabetes (Created with https://BioGDP.com (57)).

Chronic low-grade inflammation is a hallmark shared by both T2DM and osteoarthritis (26, 27). In T2DM, nutritional metabolic dysregulation, characterized by hyperglycemia and dyslipidemia, promotes the activation of inflammatory pathways within adipose tissue, pancreatic islets, and other metabolic organs (28). This systemic inflammatory state leads to increased circulating pro-inflammatory cytokines, such as tumor necrosis factor-alpha, interleukins, and C-reactive protein, which can infiltrate joint tissues (29). In osteoarthritis, these inflammatory mediators contribute to cartilage degradation, synovial inflammation, and subchondral bone changes. The persistent inflammatory milieu not only accelerates joint tissue breakdown but also exacerbates insulin resistance, creating a vicious cycle that links metabolic dysregulation with joint degeneration (30). The interplay of inflammatory processes thus serves as a central mechanism connecting T2DM and osteoarthritis pathogenesis (Figure 1).

### 3.2 Obesity as a common risk factor

Obesity, frequently resulting from nutritional metabolic dysregulation, is a significant shared risk factor for both T2DM and osteoarthritis. Excess adipose tissue, particularly visceral fat, acts as an active endocrine organ secreting adipokines and cytokines that promote systemic inflammation (31). These bioactive molecules impair insulin signaling. Simultaneously, increased body weight imposes mechanical stress on weight-bearing joints, such as the knees and hips, leading to cartilage wear and osteoarthritis (32). Moreover, adipokines like leptin and adiponectin influence cartilage metabolism and synovial inflammation, further linking obesity to joint degeneration (33, 34). Therefore, obesity serves as a nexus where nutritional metabolic dysregulation fosters both metabolic and joint pathologies through inflammatory and biomechanical pathways.

### 3.3 Nutrient metabolic changes catalyzed OA

Diabetes induces a range of metabolic alterations that predispose individuals to osteoarthritis. Hyperglycemia leads to the formation of advanced glycation end products (AGEs), which accumulate in cartilage and other joint tissues, stiffening the extracellular matrix and impairing its resilience (35, 36). These modifications compromise the biomechanical properties of cartilage, making it more susceptible to damage under normal physiological loads. Additionally, diabetes-associated mitochondrial dysfunction and oxidative stress impair chondrocyte viability and function, disrupting the maintenance of cartilage homeostasis (37, 38). Altered lipid metabolism and insulin resistance further contribute to a pro-degenerative environment within joints (39). Collectively, these metabolic disturbances foster an environment conducive to cartilage breakdown, inflammation, and joint deterioration, thereby linking abnormal nutritional metabolism in diabetes to the pathogenesis of osteoarthritis.

### 3.4 Gut microbiota dysbiosis triggers OA in T2DM

A healthy gut microbiota maintains intestinal barrier integrity and modulates immune homeostasis. Dysbiosis, characterized by reduced microbial diversity and shifts in microbial populations, can compromise the intestinal barrier, leading to increased permeability. This disruption permits translocation of microbial components such as lipopolysaccharides (LPS) into systemic circulation, triggering low-grade systemic inflammation. Such chronic inflammatory states are central to insulin resistance development in T2DM and contribute to joint tissue degradation in OA. Gut microbiota dysbiosis influences immune cell differentiation and cytokine profiles. An imbalance favoring pro-inflammatory microbial species can skew immune responses toward a Th17 and M1 macrophage phenotype, fostering an environment conducive to tissue inflammation and destruction (40, 41). In the context of OA, this immune dysregulation amplifies cartilage catabolism and synovial inflammation (42). In T2DM, immune activation contributes to pancreatic β-cell dysfunction and peripheral insulin resistance. The shared immune pathways underscore the microbiota's role in bridging metabolic and joint pathologies.

## 4 Importance of nutritional interventions for T2DM patients with OA

### 4.1 Anti-inflammatory foods

Nutritional strategies emphasizing anti-inflammatory foods play a crucial role in managing osteoarthritis (OA), especially in patients with comorbid conditions such as T2DM. Diets rich in fruits, vegetables, whole grains, fatty fish, nuts, and seeds provide bioactive compounds like omega-3 fatty acids, antioxidants, and phytochemicals that can modulate inflammatory pathways involved in OA pathogenesis (43). These foods help reduce systemic low-grade inflammation, which is a key contributor to cartilage degradation and joint pain. For instance, omega-3 fatty acids have been shown to inhibit pro-inflammatory cytokines and mediators such as leptin, which is produced by adipose tissue and has been implicated in cartilage breakdown (44). Incorporating such anti-inflammatory foods into daily diets can therefore mitigate joint inflammation, slow disease progression, and improve quality of life for OA patients. Moreover, dietary patterns like the Mediterranean diet, characterized by high intake of plant-based foods and healthy fats, have demonstrated potential in reducing inflammatory markers and supporting joint health (45).

### 4.2 Weight management and its importance

Effective weight management is fundamental in controlling osteoarthritis symptoms and progression, particularly in obese patients. Excess body weight increases mechanical load on weight-bearing joints, accelerating cartilage wear and tear (46). Beyond mechanical stress, adipose tissue secretes adipokines such as leptin and pro-inflammatory cytokines that contribute to systemic inflammation and joint degradation (47). Nutritional interventions aimed at achieving and maintaining a healthy weight can significantly reduce joint pain, improve function, and potentially delay the need for surgical interventions. Studies have shown that even modest weight loss can decrease the load on affected joints and lower systemic inflammation, thereby alleviating OA symptoms (48). For patients with T2DM, weight reduction also improves insulin sensitivity and reduces metabolic inflammation, creating a synergistic benefit for joint health (49). Therefore, integrating calorie-controlled, nutrient-dense diets with physical activity is essential for comprehensive OA management in this population (43).

### 4.3 Supplements and nutraceuticals

Nutritional supplements and nutraceuticals offer additional avenues for managing OA, particularly in addressing inflammation and cartilage repair. Omega-3 fatty acids, glucosamine, chondroitin sulfate, and certain plant-derived compounds like oleanolic acid have been investigated for their potential to modulate inflammatory responses and support joint integrity (50, 51). For example, omega-3 supplementation has been associated with reduced joint pain and stiffness, likely through its anti-inflammatory effects (52, 53). Similarly, emerging evidence suggests that nutraceuticals targeting leptin signaling and systemic inflammation can be beneficial, especially in obese OA patients where metabolic factors exacerbate joint degeneration (54). Personalized nutritional approaches, possibly guided by genetic information, may optimize the efficacy of these supplements in managing OA symptoms. However, it is important to note that while supplements can complement dietary strategies, they should be used under professional guidance to ensure safety and appropriate dosing (55). Overall, integrating nutraceuticals into a comprehensive nutritional plan can enhance joint health and improve functional outcomes in patients with OA and T2DM.

## 5 Conclusion

Nutritional metabolic dysregulation plays significant role in the development and progression of osteoarthritis among individuals with T2DM. The interconnected mechanisms—such as chronic inflammation, obesity-related biomechanical stress, and altered metabolic pathways—contribute to joint degeneration beyond traditional wear-and-tear models. Addressing metabolic disturbances through targeted nutritional interventions and lifestyle modifications can potentially mitigate osteoarthritis risk and improve patient outcomes. Recognizing the bidirectional relationship between metabolic health and joint integrity emphasizes the importance of a comprehensive approach to managing T2DM and osteoarthritis concurrently.

There is a pressing need to develop and evaluate innovative therapeutic strategies that address the metabolic underpinnings of osteoarthritis in T2DM. Future studies should explore the efficacy of targeted nutritional interventions, such as personalized diets rich in anti-inflammatory and insulin-sensitizing nutrients, alongside pharmacological agents that modulate metabolic pathways. Additionally, emerging therapies like microbiota transplantation, prebiotics, probiotics, and metabolic modulators warrant rigorous clinical testing to assess their potential in restoring metabolic balance and preventing joint degeneration (56). Integrating multi-omics data can facilitate the identification of biomarkers for treatment response, enabling more precise and effective management approaches.

To better understand the temporal relationship between nutritional metabolic dysregulation and osteoarthritis development, comprehensive longitudinal cohort studies are essential. These studies should track metabolic parameters, dietary patterns, microbiota profiles, and joint health indicators over extended periods. Such research will help clarify causative links, identify early biomarkers of joint degeneration, and determine critical windows for intervention. Moreover, stratifying participants based on metabolic phenotypes can reveal differential risks and inform personalized prevention strategies. Ultimately, these insights will guide clinical practices aimed at early detection and targeted management of osteoarthritis in individuals with T2DM.
